# Mitochondrial reprogramming in peripheral blood mononuclear cells of patients with glycogen storage disease type Ia

**DOI:** 10.1186/s12263-023-00729-y

**Published:** 2023-06-06

**Authors:** Alessandro Rossi, Antonia Assunto, Carmen Rosano, Sara Tucci, Margherita Ruoppolo, Marianna Caterino, Francesca Pirozzi, Pietro Strisciuglio, Giancarlo Parenti, Daniela Melis

**Affiliations:** 1 Department of Translational Medicine, Section of Pediatrics, University of Naples Federico II, Naples, Italy; 2 Pharmacy, Medical Center - University of Freiburg, Hugstetterstr. 55, D-79106, Freiburg, Germany; 3 Department of Molecular Medicine and Medical Biotechnology, University of Naples “Federico II”, Naples, Italy; 4 CEINGE Advanced Biotechnologies, Naples, Italy; 5 Telethon Institute of Genetics and Medicine, Pozzuoli, Italy; 6 Department of Medicine, Surgery and Dentistry “Scuola Medica Salernitana”, Section of Pediatrics, University of Salerno, Via Salvador Allende, 43 84081 Baronissi (Salerno), Italy

**Keywords:** Glycogen storage disease, Biomarker, Diet, CPT1, Fatty acid oxidation, Monitoring

## Abstract

**Background:**

Glycogen storage disease type Ia (GSDIa) is an inborn metabolic disorder caused by the deficiency of glucose-6-phospatase-α (G6Pase-α) leading to mitochondrial dysfunction. It remains unclear whether mitochondrial dysfunction is present in patients’ peripheral blood mononuclear cells (PBMC) and whether dietary treatment can play a role. The aim of this study was to investigate mitochondrial function in PBMC of GSDIa patients.

**Methods:**

Ten GSDIa patients and 10 age-, sex- and fasting-time matched controls were enrolled. Expression of genes involved in mitochondrial function and activity of key fatty acid oxidation (FAO) and Krebs cycle proteins were assessed in PBMC. Targeted metabolomics and assessment of metabolic control markers were also performed.

**Results:**

Adult GSDIa patients showed increased *CPT1A*, *SDHB*, *TFAM*, *mTOR* expression (*p* < 0.05) and increased VLCAD, CPT2 and citrate synthase activity in PBMC (*p* < 0.05). VLCAD activity directly correlated with WC (*p* < 0.01), BMI (*p* < 0.05), serum malonycarnitine levels (*p* < 0.05). CPT2 activity directly correlated with BMI (*p* < 0.05).

**Conclusion:**

Mitochondrial reprogramming is detectable in PBMC of GSDIa patients. This feature may develop as an adaptation to the liver enzyme defect and may be triggered by dietary (over)treatment in the frame of G6Pase-α deficiency. PBMC can represent an adequate mean to assess (diet-induced) metabolic disturbances in GSDIa.

**Supplementary Information:**

The online version contains supplementary material available at 10.1186/s12263-023-00729-y.

## Introduction

Glycogen storage disease type Ia (GSDIa) (MIM# 232200) is an inborn disorder of carbohydrate metabolism due to *G6PC* gene variants. *G6PC* gene is expressed in the liver, kidney and intestine and encodes for the enzyme glucose 6-phosphatase-α (G6Pase-α). G6Pase-α deficiency leads to impairment of both glycogenolysis and gluconeogenesis and is associated with accumulation of glycogen and fat in the liver and kidney [1]. Hepatomegaly, fasting hypoglycemia, elevated lactate, metabolic acidosis, hyperlipidemia, hyperuricemia and elevated transaminases are common manifestations of GSDIa [2]. Dietary management is the cornerstone of treatment for GSDIa patients. It is based on frequent feedings, including uncooked cornstarch (UCCS)/Glycosade and/or continuous gastric drip-feeding (CNGDF). Despite dietary treatment, GSDIa patients may develop various (long-term) complications, including liver adenomas and chronic kidney disease (CKD) [3]. Medical treatment can be employed to correct secondary metabolic disturbances or delay disease complications [4].

Reduced mitochondria number in GSDIa patients’ liver was first reported in 1980 [5]*.* More recently, reduced mitochondrial content, abnormal mitochondrial morphology, impaired mitochondrial respiration, and disturbed TCA cycle function have been demonstrated in the liver of *G6pc*^*−/−*^ mice [6]. Lower concentration of malate has been found in fibroblasts from one GSDIa patient [7]. Decreased succinate dehydrogenase and NADH dehydrogenase activity have been demonstrated in peripheral lymphocytes from GSDI patients [8]. We have previously reported on indirect markers of tricarboxylic acid (TCA) cycle and fatty acid oxidation (FAO) overload in GSDIa patients’ urine and plasma, respectively [9]. Yet, it remains unclear whether changes in mitochondrial function can be systematically detected in a minimally invasive manner in GSDIa patients’ peripheral blood mononuclear cells (PBMC). It also remains unknown whether dietary treatment can concur to mitochondrial dysfunction in GSDIa.

The aim of the present study was to investigate mitochondrial function in PBMC of GSDIa patients.

## Methods

### Study design

This was a cross-sectional pilot study. The study protocol was in accordance with the Italian regulations on privacy protection and with the Helsinki Doctrine for Human Experimentation of 1975 as revised in 2013. All participants (or participants’ parents) provided written informed consent prior to inclusion in the study. 10 GSDIa patients were enrolled and compared to 10 age and sex matched controls (Supplemental figure 1). The study was designed to assess in all study participants: 1) the expression of a subset of genes implicated in mitochondrial activity in PBMC, 2) the activity of a subset of proteins involved in mitochondrial FAO and TCA cycle in PBMC and 3) the levels of metabolites associated with FAO or TCA cycle in serum and urine, respectively. Additionally, biochemical markers of metabolic control were assessed in GSDIa patients. All samples were collected in a pre-prandial/fasted state. To minimize the possible effect of diet and fasting time, each adult control (*n* = 6, control group 1 (C1)) was asked to follow a “GSDIa- like” dietary regimen for 7 days and subsequently have blood collection at the same fasting time as his/her matched patient. “GSDIa- like” dietary regimen followed by each control included same meal schedule, same Kcal/day and same % of daily macronutrients (carbohydrates, lipid, protein) as compared to his/her matched patient; UCCS was substituted with an equivalent amount of natural complex carbohydrates (e.g., pasta, bread, potatoes). As GSDIa patients are dependent on frequent feedings (including overnight) due to G6Pase-α deficiency, to further assess the effect of (short) fasting time, analysis were also performed on one additional blood sample collected after 8-h fasting in adult controls (*n* = 6, control group 2 (C2)). Due to the difficulty of following a “GSDIa-like” dietary regimen in children, each pediatric control (*n* = 4) had blood sampling under standard dietary regimen after the same fasting time of his/her age and sex matched patient. Similar to adult controls, in whom fasting time was matched between each patient and his/her matched control, this group is also indicated as C1 (*n* = 4). Whether C1 refers to adult or pediatric controls is clarified in the text and/or figures and/or tables at each occurrence. Blood and urine samples were collected between 8–11 am depending on each patient’s meal schedule, in order to have the same fasting time for GSDIa patients, C1 and pediatric controls. Blood samples were collected at 8 am for C2.

### Subjects

Participants were recruited over a 12-month period. Ten GSDIa patients (6 males and 4 females, median age 20.50 ± 9.44 years, age range: 5.20–34.25 years) were enrolled. Based on their age, patients were classified as children (age < 16 years, *n* = 4) or adults (age ≥ 16 years, *n* = 6). The diagnosis of GSDIa was based on mutation analysis of the *G6PC* gene. Mean age at diagnosis was 0.79 ± 0.21 years. 1 GSDIa patient had GH deficiency and was treated with rhGH. 2 GSDIa patients were treated with ACE-inhibitors. 2 GSDIa patients were treated with both ACE-inhibitors and fenofibrate.

All patients were on dietary treatment (Supplemental Table 1). Each patient received uncooked cornstarch (UCCS), CNGDF or a combination of the two. Dietary regimens varied among different patients according to their families’ requests and attitudes. Carbohydrate intake was 8.82 ± 3.90 g/kg/day. The dose of UCCS was 1.0–1.5 g/kg/meal. Nocturnal carbohydrate intake was 2.21 ± 0.55 g/kg in patients receiving UCCS and 3.59 ± 1.41 g/kg in patients receiving CNGDF. In patients receiving CNGDF, the rate of glucose administration varied according to the patients’ age, ranging from 4 to 10 mg/kg/min.

Ten age- and sex-matched subjects with normal random blood glucose and no history of hypoglycemia were included as healthy control participants.

### Clinical and biochemical parameters

The following clinical parameters were recorded: body mass index (BMI), waist circumference (WC). Biochemical markers of metabolic control included: serum glucose, triglycerides (TG), cholesterol, lactate, uric acid, aspartate aminotransferase (AST), alanine aminotransferase (ALT).

### Gene expression analysis

Relative expression of a subset of genes implicated in mitochondrial activity was assessed using predeveloped TaqMan assay primers and probes (Thermo Fisher Scientific, Waltham, MA, USA). mRNA isolation was carried out as previously described [10]. One µg of RNA was reverse transcribed in a 20 μl reaction mixture using the High Capacity cDNA Reverse Transcription kit (Applied Biosystems, Foster City, CA, USA) and the resulting cDNA was diluted tenfold in 180 μl nuclease-free water. To detect the expression levels of all investigated genes, real-time quantitative PCR (RT-qPCR) was performed with TaqMan Gene Expression PCR Master Mix (Applied Biosystems, Foster City, CA, USA) on ABI 7900 Real-Time PCR instrument (Thermo Fisher Scientific), according to manufacturer’s instructions. Beta-2-microglobulin (*B2M*) and hypoxanthine phosphoribosyltransferase 1 (*HPRT1*) housekeeping genes were used as internal controls. The data were analyzed with the SDS relative quantification software version 1.2.1 (Thermo Fisher Scientific). Relative quantification was performed using the Pfaffl method [10]. Selected genes included:

- 1) genes involved in mitochondrial function: carnitine palmitoyltransferase 1A (*CPT1A*), succinate dehydrogenase complex flavoprotein subunit A (*SDHA*), succinate dehydrogenase complex flavoprotein subunit B (*SDHB*), succinate dehydrogenase complex flavoprotein subunit C (*SDHC*), ATP-citrate lyase (*ACLY*), nicotinamide phosphoribosyltransferase (*NAMPT*);
- 2) genes involved in mitochondrial biogenesis: nuclear respiratory factor 1 (*NRF1*), transcription factor A mitochondrial (*TFAM*), uncoupling protein 3 (*UCP3*), peroxisome proliferative activated receptor, gamma, coactivator 1, Alpha (*PCG1α*), sirtuin 1 (*SIRT1*), sirtuin 2 (*SIRT2*);
- 3) genes involved in intracellular sensing: mechanistic target of rapamycin (*mTOR*), AKT serine/threonine kinase 1 (*AKT1*), AKT serine/threonine kinase 2 (*AKT2*), AKT serine/threonine kinase 3 (*AKT3*).

A full list of genes and TaqMan assays used in this study is reported in Supplemental Table 2.

### Enzyme assays

Proteins involved in mitochondrial FAO and TCA cycle included medium-chain acyl-CoA dehydrogenase (MCAD), very long-chain acyl-CoA dehydrogenase (VLCAD), citrate synthase (CS) and carnitine palmitoyltransferase II (CPT2). Functional testing was performed as previously reported [11–14].

### Metabolomics studies

Blood and urine samples were collected to measure the levels of 26 Acylcarnitines (ACs) and 76 urine organic acids (UOA). Whole blood and serum were collected in serum tubes and stored between 2 and 8 °C. Fresh urine samples were collected in a test tube and stored frozen. All reagents and internal standards used for LC–MS/MS and for GC–MS were as previously described [15].

The ACs, extracted and derivatized to butyl esters as previously described [15, 16], were analyzed on an API 4000 triple quadrupole mass spectrometer (Applied Biosystems-Sciex, Toronto, Canada) coupled with the high performance liquid chromatograph Agilent 1100 series (Agilent Technologies, Waldbronn, Germany).

Creatinine concentration of urine samples was determined by using automatic analysis system BM/Hitachi 904. Organic acids were extracted from urine and analysed on a GC–MS system including an Agilent 7890A (Agilent Technologies, Santa Clara, CA, USA) gas chromatograph and an Agilent 5975C mass spectrometer. Extraction and analysis were performed as previously described [17]; the concentration of organic acids was normalized to the creatinine concentration of the urine sample and expressed as mmol organic acid/mol creatinine.

### Statistical analysis

All data in the text or shown in the figures are expressed as mean ± SE. Statistical analysis was performed using Statistical Package for Social Science (SPSS 10 for Windows Update; SPSS Inc., Chicago, Illinois, USA). Since this was a pilot study, no formal sample size calculation was performed. The comparisons between numerical variables were performed by Student’s t-test corrected for Fisher’s exact test. The normality of the distribution was checked by the Shapiro–Wilk test. Due to small sample size (*n* = 4), the normality of the distribution could not be checked in children; thus, it was assumed that these data points were non normally distributed. Correlation study was performed by Spearman's rank correlation. Statistical significance was set at *p* < 0.05.

## Results

### Clinical and biochemical parameters

Clinical and biochemical parameters are summarized in Table 1. As expected, GSDIa patients showed higher BMI, WC, serum cholesterol, TG, lactate, uric acid, AST, ALT levels compared to controls [9, 18, 19].

**Table 1:** Clinical and biochemical parameters of GSDIa patients and controls

	**GSDIa**		**Controls**		**Significance**
	Mean	SE	Mean	SE	p
BMI	23.4	1.0	20.0	1.1	**< 0.05**
WC^a^ (cm)	90.1	3.2	73.4	2.6	**< 0.01**
Glucose (mg/dl)	98.1	9.8	82.7	2.7	> 0.05
Lactate (mmol/l)	3.6	0.6	1.5	0.1	**< 0.01**
Cholesterol (mg/dl)	250.6	26.7	152.2	6.8	**< 0.01**
TG (mg/dl)	665.3	219.3	125.5	10.5	**< 0.05**
Uric acid (mg/dl)	5.6	0.5	4.0	0.2	**< 0.05**
AST (U/l)	59.0	13.7	21.7	1.3	**< 0.05**
ALT (U/l)	61.2	12.3	18.8	1.3	**< 0.01**

*ALT* Alanine aminotransferase, *AST* Aspartate aminotransferase, *BMI* Body mass index, *SE* Standard error, *TG* Triglycerides, *WC* Waist circumference

^a^7 GSDIa patients and 7 controls

### Abnormalities in mitochondrial metabolism are present in serum and urine of GSDIa patients

**Fig. 1:**
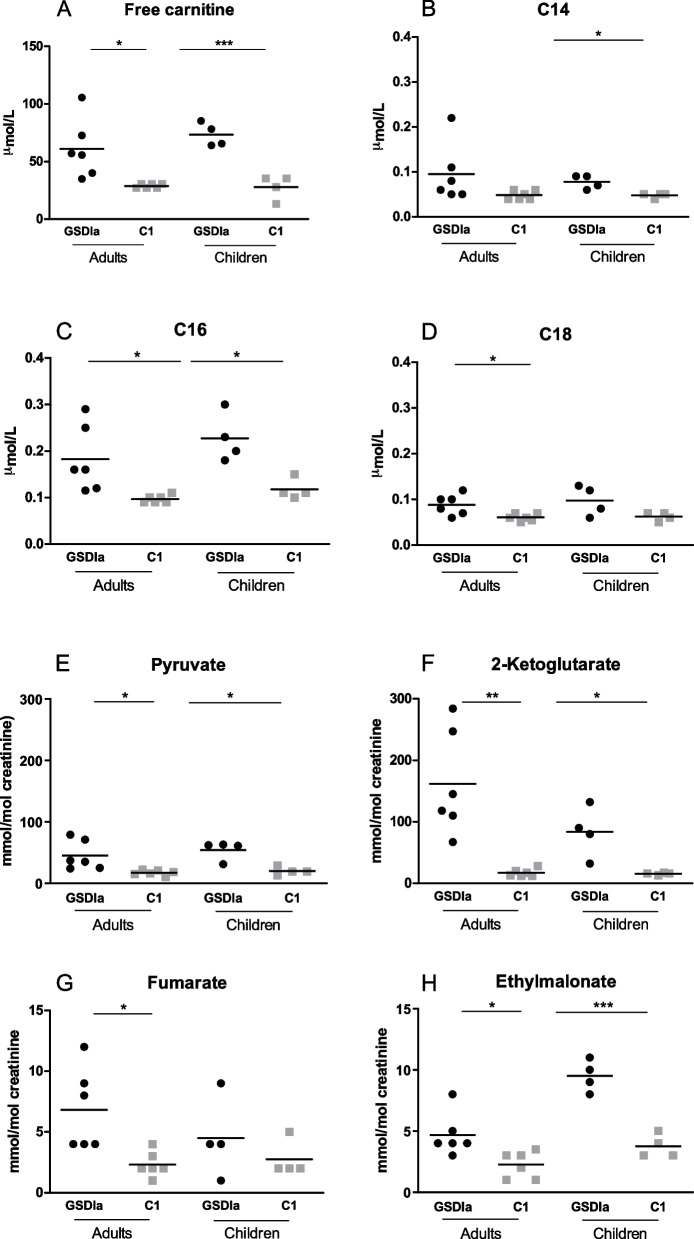
Serum acylcarnitines (**A**-**D**) and urine organic acids (**E**–**H**) in GSDIa patients and control groups. For all metabolites mean value is shown for each group of participants. C1: controls who had blood collection after the same fasting time as their matched patients (adults: *n* = 6, children: *n* = 4). C14: miristoylcarnitine; C16: palmitoylcarnitine; C18: stearoylcarnitine * *p* < 0.05; ** *p* < 0.01; ***p < 0.001

In line with previous report [9], higher serum free carnitine levels were found both in adult GSDIa patients and children compared to age-, sex- and fasting time-matched controls (C1) (Fig. 1A). Adult GSDIa patients also showed higher serum palmitoylcarnitine and stearoylcarnitine concentrations compared to C1. GSDIa children showed higher miristoylcarnitine and palmitoylcarnitine concentrations compared to C1 (Fig. 1B-D). Higher urine pyruvate, ethylmalonate and 2-ketoglutarate excretion were found both in adult GSDIa patients and children compared to C1. Adult GSDIa patients also showed increased urine pyruvate, 2-ketoglutarate, fumarate, ethylmalonate excretion compared to C1 (Fig. 1E-H). These data confirmed that abnormalities in mitochondrial metabolism are present in extra-hepatic tissues in GSDIa.

### Expression of genes involved in mitochondrial biogenesis, function and intracellular sensing is increased in PBMC of adult GSDIa patients

**Fig. 2:**
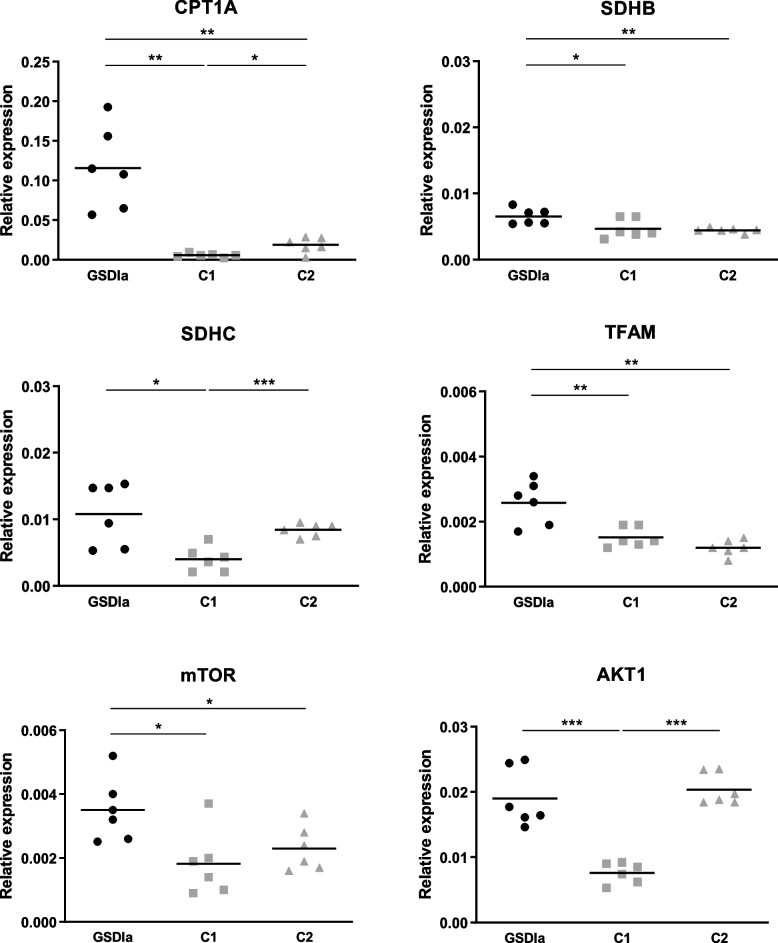
Gene expression analysis in adult GSDIa patients (*n* = 6) and control groups. For all genes mean value is shown for each group of participants. C1: adult controls who had blood collection after the same fasting time as their matched patient (*n* = 6). C2: adult controls who had blood collection after 8-h fasting (*n* = 6).* *p* < 0.05; ** *p* < 0.01; ***p < 0.001

To ascertain whether mitochondrial dysfunction was also detectable in PBMC of GSDIa patients’, the expression of a subset of key genes involved in mitochondrial activity was assessed. Adult GSDIa patients showed significantly higher *CPT1A, SDHB, SDHC, TFAM, mTOR* and *AKT1* expression compared to age-, sex-, fasting time- and diet-matched controls (C1) and significantly increased *CPT1A, SDHB, TFAM,* and *mTOR* expression compared to age-, sex- and not fasting-time/diet-matched controls (C2). *CPT1A, SDHC* and *AKT1* expression was significantly higher in C2 compared to C1 (Fig. 2). Despite not statistically significant, a trend towards increased *CPT1A* expression in GSDIa children compared to C1 was observed (*p* = 0.08, supplemental figure 2).

### Fatty acid oxidation is upregulated in PBMC of GSDIa patients

**Fig. 3:**
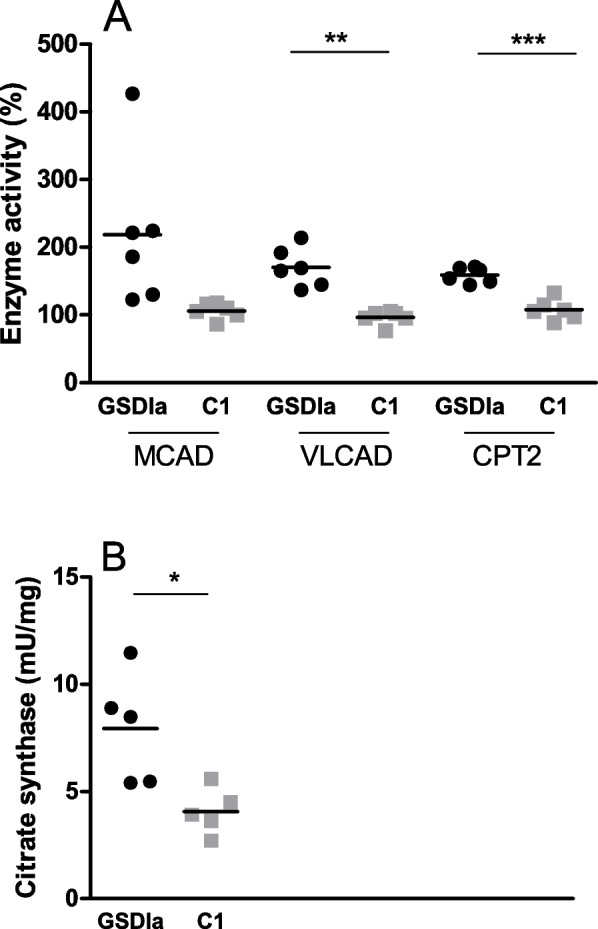
FAO enzymes (**A**) and citrate synthase (**B**) activity in adult GSDIa patients (*n* = 6) and controls. FAO enzyme activity is presented as % activity compared to the mean calculated on a pool of healthy subjects. C1: adult controls who had blood collection after the same fasting time as their matched patients (*n* = 6). For all proteins mean value is shown for each group of participants. * *p* < 0.05; ** *p* < 0.01; ***p < 0.001

Since fatty acid oxidation (FAO) and TCA cycle are major mitochondrial functions, we investigated the activity of a subset of proteins involved in these pathways in PBMC. Increased MCAD activity was found in all adult GSDIa patients (range 122.7–426.9%) (Fig. 3A) and 2/4 GSDIa children (range 62.4–147.1%) (Supplemental Fig. 3). Similarly, palmitoyl-CoA oxidation rate as indicator of VLCAD activity was increased in all adult GSDIa patients (range 136.8–214%) (Fig. 3A) and 2/4 children (range 64.9–156%) (Supplemental figure 3). Increased CPT2 activity was found in all adult GSDIa patients (range 144.2–171.1%) (Fig. 3A) and 3/4 children (range 88.8–143.4%) (Supplemental Figure 3). Adult GSDIa patients showed increased VLCAD and CPT2 activity compared to age-, sex-, fasting time- and diet-matched controls (C1) (Fig. 3A). CS activity was assessed in 8/10 GSDIa patients and 8/10 controls (10 adults and 6 children). Adult GSDIa patients showed higher CS activity compared to C1, suggesting mitochondrial proliferation (Fig. 3B). No significant difference in CS activity was observed between children with GSDIa and C1 (Supplemental Figure 3).

### Mitochondrial markers correlate with dietary data in GSDIa patients

**Fig. 4:**
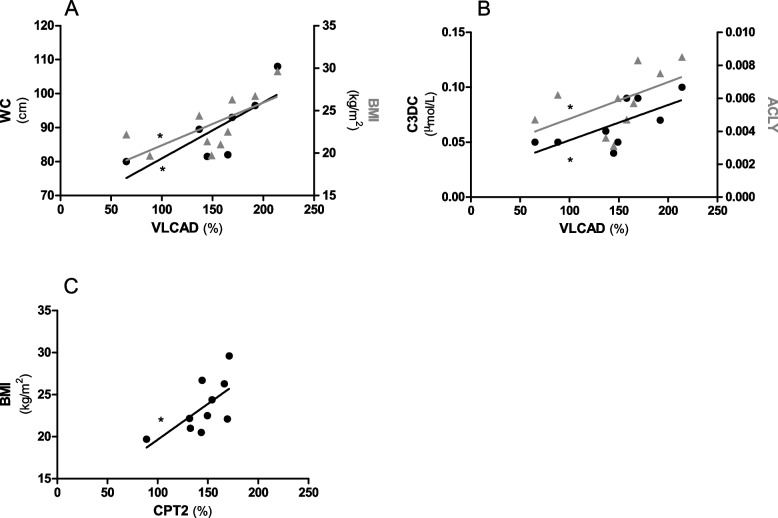
Correlation analysis in GSDIa patients. **A** Correlation between VLCAD activity and waist circumference (WC, black circles, ρ = 0.89, *n* = 7 subjects) and BMI (grey triangles, ρ = 0.77, *n* = 10 subjects). **B** Correlation between VLCAD activity and serum malonylcarnitine (C3DC, black circles, ρ = 0.67, *n* = 9 subjects) and ACLY mRNA levels (grey triangles, ρ = 0.66, *n* = 10 subjects). **C** Correlation between CPT2 activity and BMI (black circles, ρ = 0.64, *n* = 10 subjects). * *p* < 0.05

In GSDIa patients a direct correlation was found between: a) VLCAD activity and WC (*p* < 0.01), BMI (*p* < 0.05) (Fig. 4A), serum malonycarnitine levels (*p* < 0.05) and *ACLY* mRNA levels (*p* < 0.05) (Fig. 4B) and b) CPT2 activity and BMI (*p* < 0.05) (Fig. 4C). A direct correlation between VLCAD activity and WC (*p* < 0.05), BMI (*p* < 0.05), and *ACLY* mRNA levels (*p* < 0.05) as well as between CS activity and *ACLY* expression (*p* < 0.05) was found when analyzing adult GSDIa patients separately (Supplemental Figure 4). None of the above-mentioned correlation was found in GSDIa children, in whom a direct correlation between ACLY expression level and serum AST (*p* < 0.01) and ALT (*p* < 0.05) was observed (Supplemental Figure 5).

**Fig. 5:**
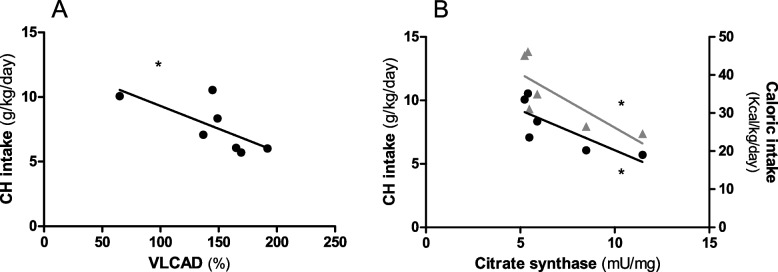
Correlation analysis in GSDIa patients not receiving continuous nocturnal gastric drip feeding (CNGDF). **A** Correlation between VLCAD activity and daily carbohydrate (CH) intake (black circles, ρ = -0.79, *n* = 7 subjects). **B** Correlation between citrate synthase activity and daily carbohydrate (CH) intake (black circles, ρ = -0.89, *n* = 6 subjects) and daily caloric intake (grey triangles, ρ =—0.89, *n* = 6 subjects). * *p* < 0.05

In GSDIa patients who did not receive CNGDF an inverse correlation was found between: a) VLCAD activity and daily carbohydrate intake (ρ = -0.79, *p* < 0.05) (Fig. 5A) and b) CS activity and daily carbohydrate intake (ρ = -0.89, *p* < 0.05) and daily kcal intake (ρ =-0.89, *p* < 0.05) (Fig. 5B).

## Discussion

To the best of our knowledge this is the first study to systematically assess mitochondrial activity in PBMC of GSDIa patients. The expression of a subset of genes implicated in mitochondrial function and biogenesis and intracellular sensing as well as the activity of a subset of proteins involved in mitochondrial FAO and TCA cycle were evaluated in GSDIa patients and age-, and gender- matched healthy controls (C2). To gather information on the possible relationship between mitochondrial (dys)function and dietary treatment, genomic and enzyme activity analysis were also performed in adult age-, gender-, fasting time- and diet-matched controls (C1). Targeted metabolomics was also performed on participants' serum and urine. Adult GSDIa patients showed significantly higher *CPT1A, SDHB, SDHC, TFAM, mTOR* and *AKT1* expression compared to age-, sex-, fasting time- and diet-matched controls (C1) and significantly increased *CPT1A, SDHB, TFAM,* and *mTOR* expression compared to age-, sex- and not fasting-time/diet-matched controls (C2). *CPT1A, SDHC* and *AKT1* expression was significantly higher in C2 compared to C1. Increased VLCAD, CPT2 an citrate synthase activity were found in PBMC of adult GSDIa patients. A direct correlation was found between VLCAD and WC, BMI and serum malonylcarnitine levels in adult GSDIa patients. A direct correlation between CPT2 activity and BMI was also found. Accumulation of free carnitine and long-chain acylcarnitines as well as increased urine excretion of various TCA cycle metabolites were found in GSDIa patients.

Metabolomics findings were consistent with previous research reporting accumulation of various serum acylcarnitines and increased urine excretion of various TCA cycle metabolites in GSDIa patients [8, 20]. Notably, the degree of increased urinary excretion was higher for downstream mitochondrial metabolites (i.e. 2-ketoglutarate, fumarate, ethylmalonate) than pyruvate. This likely reflects the multiple pathways towards which excess pyruvate is hijacked (i.e. reduction to lactate, acetyl-CoA synthesis, pyruvate/malate shuttle). Adult GSDIa patients also showed increased *CPT1A*, *SDHB*, *TFAM*, *mTOR* expression in PBMC. Because the activation of *mTOR* mediates the fatty acid metabolic reprogramming with upregulation of mitochondrial degradation via PPARα [21, 22] we were not surprised to observe a parallel increase of the corresponding enzymes VLCAD, CPT2 and CS activity in PBMC of GSDIa patients. These data strongly suggest that mitochondrial reprogramming associated with increased oxidative processes for energy production are detectable in PBMC of GSDIa patients.

*CPT1A*, CPT2 and VLCAD play a key role in the mitochondrial FAO. The ubiquitously expressed *CPT1A* gene encodes for the CPT1, which is located in the mitochondrial outer membrane and catalyzes the rate-limiting step of the mitochondrial FAO [23, 24]. As CPT1 is inhibited by the cytosolic accumulation of malonyl-CoA [25, 26], a reduction of liver mitochondrial FAO has been postulated in in GSDIa [27]. Consistently, studies in *G6pc*^*−/−*^ mice demonstrated reduced *cpt1a* and *acadvl* (i.e. the gene encoding for the VLCAD) expression in the liver (and kidney), yet associated with normal *cpt2* expression [28–31]. CPT1 protein over-representation was found in the liver of older *G6pc*^*−/−*^ mice [32]. These data may suggest that, although the transport of acyl-residues across the membrane seems to be inhibited as indicated by the enhanced concentration of CPT1 protein, the intramitochondrial activation of fatty acids for FAO appears to be maintained. The upregulated activity of mitochondrial FAO in PBMC from GSDIa patients reported in this work corroborates this hypothesis. We hypothesize that enhanced expression of *CPT1A* accompanied by the increased VLCAD and CPT2 activity in PBMC may likely represents a mechanism to counter-act malonyl-CoA-induced inhibition of hepatic CPT1 activity in response to high circulating fatty acids [25]. A possible correlation between (increased) expression of genes involved in FAO and residual G6Pase-α activity has been recently reported in *G6pc*^*−/−*^ mice [33], supporting this hypothesis. Since 8/10 GSDIa patients carried *G6PC* mutations which completely abolished G6Pase-α activity [1], a correlation between FAO upregulation and estimated G6Pase-α residual activity could not be assessed in the present study.

Besides the enzyme defect *per se*, mitochondrial FAO can be affected by the diet. Plasma fatty acids levels from obese individuals are usually elevated [34]. Increased *CPT1A* expression was reported in PBMC from obese individuals [35, 36] and lymphocytes from patients with type 2 diabetes [37]. Significantly different *CPT1A* expression was found between the two control groups in the present study (Fig. 2), suggesting a role of dietary treatment in FAO upregulation in GSDIa patients. We hypothesize that an increase in mitochondrial FAO in response to dietary (over) treatment can occur up to a certain threshold in GSDIa patients. Beyond this limit mitochondrial compensation is exceeded and FAO becomes overloaded. This hypothesis is line with experimental data from a computational model of mitochondrial FAO, showing increased FAO flux occurring up to 50 mM palmitoyl-CoA followed by a subsequent decreased flux (together with acyl-CoA accumulation) when the concentration of palmitoyl-CoA was further increased [38]. We speculate that enhanced mitochondrial FAO in PBMC may represent an adaptive endogenous response to counteract lipid overload and lipotoxicity (secondary to defective liver mitochondrial FAO and increased lipogenesis) in GSDIa patients otherwise concurring to the development of metabolic syndrome and insulin-resistance [8, 17]. Similarly to type 2 diabetes, increased lipid flux through the CPT1 may lead to FAO overload over time in GSDIa with the accumulation of long-chain acylcarnitines in PBMC [37]. In this small sample size study, adaptations to the various dietary regimens (i.e. UCCS/Glycosade and CNGDF) could not be compared. As decreased mitochondrial FAO contributes to CKD progression [39] we cannot rule out that such adaptive response may also occur in other tissues possibly playing a role in the development of long-term complications in GSDIa.

Consistent with our hypothesis it is not surprising that increased *mTOR, TFAM and SDHB* expression as well as increased CS activity were found in PBMC of adult GSDIa patients. *mTOR* activation concurs to the detrimental effects of fatty acids in various cell types [40, 41]. Particularly, obesity and type 2 diabetes results in *mTOR* hyperexpression in various tissues [42], including PBMC [43]. *TFAM* is a key regulator of mitochondrial DNA replication and transcription. Its expression is modulated by a number of factors, including sirtuins and *PCG1α* [44]. Previous studies showed that the role of *TFAM* in regulating mitochondrial fuel metabolism and ROS emission is tissue specific [45, 46]. Specifically, muscle-specific *TFAM* overexpression was associated with increased FAO, CS and TCA cycle activity in mice fed a high-fat diet [47]. Notably, decreased *TFAM* expression and unchanged *mTOR* mRNA levels were found in the liver *G6pc*^*−/−*^ mice [5, 48]. Data from the present study support the hypothesis that extra-hepatic tissue may deploy an adaptive (i.e. opposite) mitochondrial reprogramming in response to the metabolic abnormalities secondary to the liver G6Pase-α defect. m-TOR modulator AMPK signaling has been shown to be downregulated in the liver of GSDIa mice [48]. Further studies assessing AMPK signaling in GSDIa patients are worthy.

Although no significant difference in mean values was found, higher *CPT1A* mRNA levels as well as increased VLCAD and CPT2 activity were found in 2/4, 3/4 and 2/4 GSDIa children compared to their matched controls, respectively. These data support the hypothesis that FAO upregulation in GSDIa patients’ PBMC arises from the combination of G6Pase-α deficiency and (long-term) dietary treatment. Additional studies with larger children’s groups are warranted.

The study has some potential limitations. First, a small number of patients with GSDIa was included. Since this was a pilot study, no formal sample size calculation was performed. The limited sample size could have contributed to the lack of statistical significance, especially in children. Second, it is unclear whether the study population adequately reflects the large clinical and biochemical heterogeneity displayed by patients with GSDIa. Particularly, 8/10 GSDIa patients carried the p.Arg83Cys variant (which completely abolish G6Pase-α function). Therefore, effect of genotype on mitochondrial activity could not be assessed. Third, only 3/10 GSDIa patients received CNGDF with the remainders receiving UCCS/Glycosade in the present study. Thus, contribution of mitochondrial adaptation to the various dietary regimens (i.e. CNGDF and UCCS/Glycosade) to mitochondrial reprogramming could not be assessed. Fourth, downstream and side mitochondrial pathways (e.g. AMPK, autophagy) were not investigated in the present study.

In conclusion, we showed that mitochondrial reprogramming (i.e., FAO and TCA cycle hyperactivation) is detectable in PBMC from GSDIa patients as opposed to previously reported liver findings. We hypothesize that mitochondrial reprogramming in PBMC occurs as an adaptation to metabolic abnormalities secondary to the G6Pase-α defect in the liver. Likely, dietary (over)treatment (i.e. frequent feedings, excess UCCS/Glycosade) may trigger mitochondrial reprogramming in the frame of G6Pase-α deficiency-induced pathologic environment. PBMC can represent an adequate mean to assess secondary (diet-induced) disturbances in GSDIa in a minimally invasive way [49].

## Supplementary Information

**Additional file 1:** **Supplemental figure 1.** Study design and subjects. FAO: fatty acid oxidation; PBMC: peripheral blood mononuclear cells; TCA: tricarboxylic acid * GSDIa patients and C1.

**Additional file 2:** **Supplemental figure 2.** CPT1A expression in GSDIa childrenand pediatric controls. Each control had blood sampling under standard dietary regimen after the same fasting time of his/her age and sex matched patient. Mean value is shown for each group of participants. *p*=0.08.

**Additional file 3:** **Supplemental figure 3.**FAO enzymes in GSDIa children. FAO enzyme activity is presented as % activity compared to the mean calculated on a pool of healthy subjects.Citrate synthase activity in GSDIa childrenand healthy controls. For all proteins mean value is shown for each group of participants. C1: pediatric controls who had blood collection after the same fasting time as their matched patients.

**Additional file 4:** **Supplemental figure 4.** Correlation analysis in adult GSDIa patients.Correlation between VLCAD activity and waist circumferenceand BMI.Correlation between VLCAD activity and ACLY mRNA levels.Correlation between citrate synthase activity and ACLY mRNA levels. * *p*< 0.05.

**Additional file 5:** **Supplemental figure 5.** Correlation between ACLY mRNA levels and serum ASTand ALT. *p< 0.05; **p< 0.01.

**Additional file 6:** **Supplemental table 1. **Clinical information of GSDIa patients.

**Additional file 7:** **Supplemental table 2. **The list of TaqMan assays used for gene expression analysis by real-time quantitative PCR.
