# Supplementary material for: Mitochondrial reprogramming in peripheral blood mononuclear cells of patients with glycogen storage disease type Ia
Source: Genes Nutr. 2023 Jun 6;18:10. doi: 10.1186/s12263-023-00729-y (PMC10245432; doi:10.1186/s12263-023-00729-y)
Supplement: Supplementary file 1 — Additional file 1: Supplemental figure 1. Study design and subjects. FAO: fatty acid oxidation; PBMC: peripheral blood mononuclear cells; TCA: tricarboxylic acid * GSDIa patients and C1. [file 12263_2023_729_MOESM1_ESM.pdf]

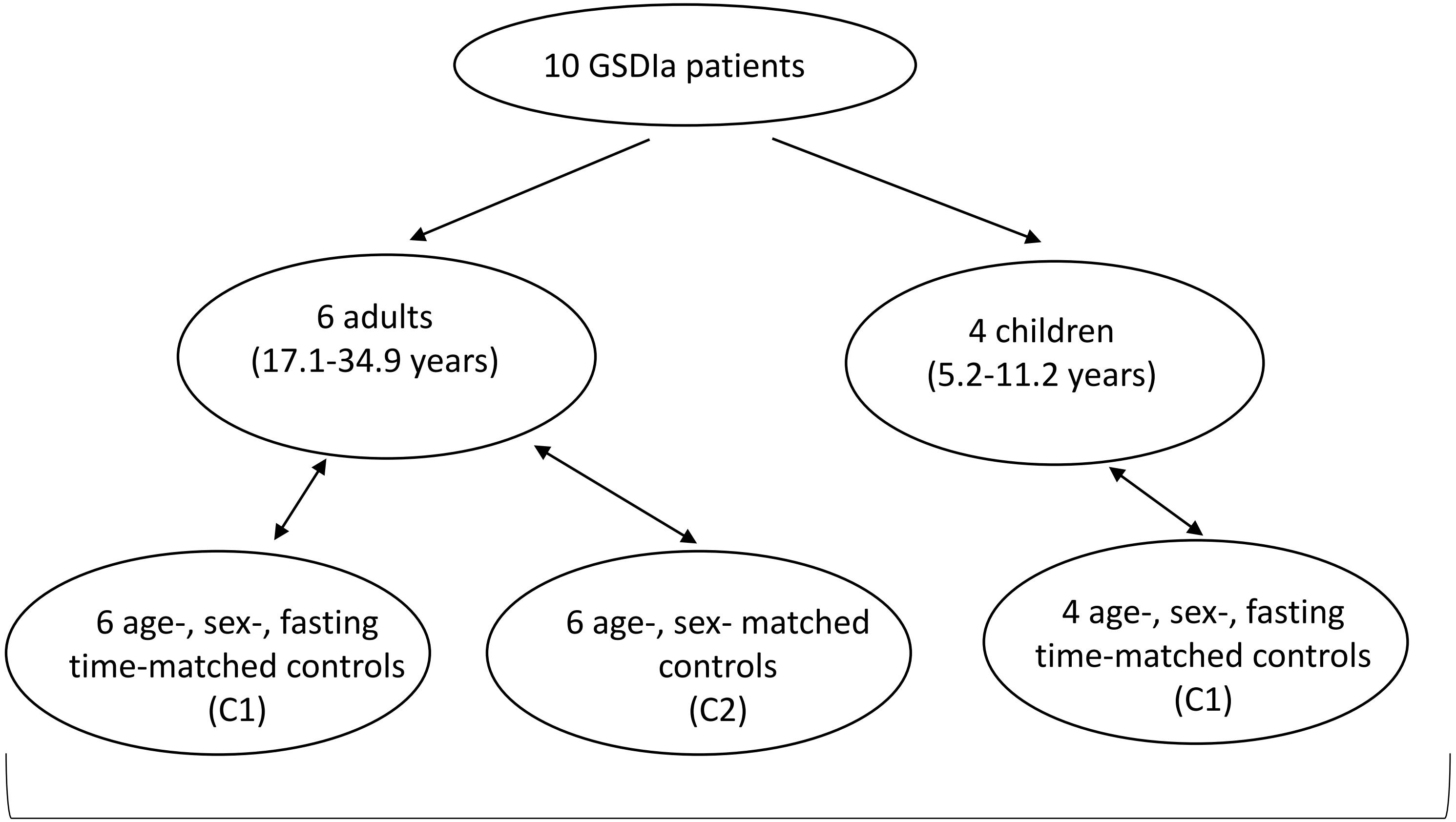

- **Gene expression analysis** (mitochondrial activity)
  PBMC
- **Enzyme analysis** (mitochondrial FAO, TCA cycle)*
  PBMC
- **Metabolomics***
  Serum, urine
