# Supplementary figures and images for: Mitochondrial reprogramming in peripheral blood mononuclear cells of patients with glycogen storage disease type Ia
Source: Genes Nutr. 2023 Jun 6;18:10. doi: 10.1186/s12263-023-00729-y (PMC10245432; doi:10.1186/s12263-023-00729-y)

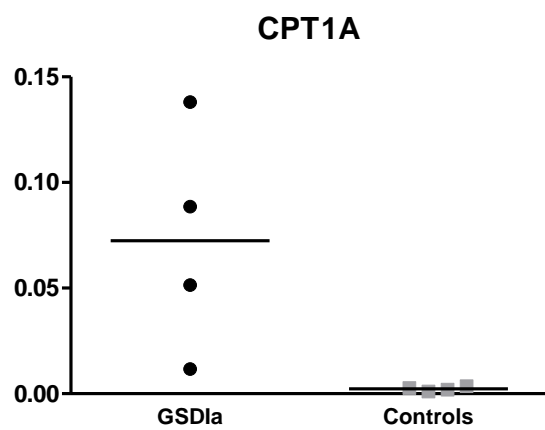

Supplemental Figure 2.

Supplement: Supplementary file 2 — Additional file 2: Supplemental figure 2. CPT1A expression in GSDIa childrenand pediatric controls. Each control had blood sampling under standard dietary regimen after the same fasting time of his/her age and sex matched patient. Mean value is shown for each group of participants. p=0.08. [file 12263_2023_729_MOESM2_ESM.pdf]

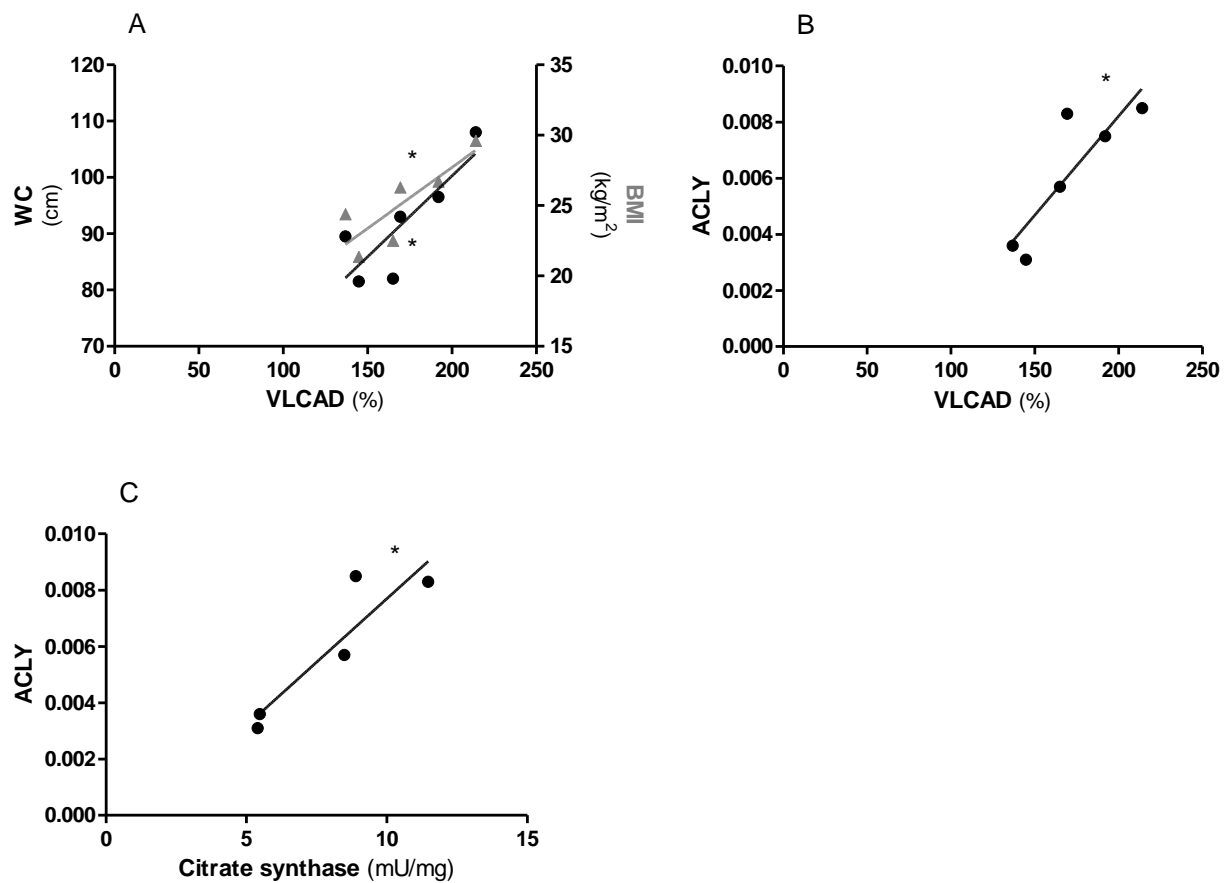

Supplemental figure 4.

Supplement: Supplementary file 4 — Additional file 4: Supplemental figure 4. Correlation analysis in adult GSDIa patients.Correlation between VLCAD activity and waist circumferenceand BMI.Correlation between VLCAD activity and ACLY mRNA levels.Correlation between citrate synthase activity and ACLY mRNA levels. * p< 0.05. [file 12263_2023_729_MOESM4_ESM.pdf]

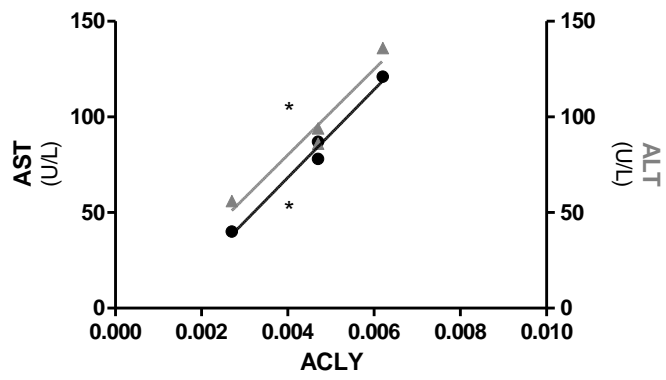

Supplemental figure 5.

Supplement: Supplementary file 5 — Additional file 5: Supplemental figure 5. Correlation between ACLY mRNA levels and serum ASTand ALT. *p< 0.05; **p< 0.01. [file 12263_2023_729_MOESM5_ESM.pdf]
