# Supplementary material for: Mitochondrial reprogramming in peripheral blood mononuclear cells of patients with glycogen storage disease type Ia
Source: Genes Nutr. 2023 Jun 6;18:10. doi: 10.1186/s12263-023-00729-y (PMC10245432; doi:10.1186/s12263-023-00729-y)
Supplement: Supplementary file 3 — Additional file 3: Supplemental figure 3.FAO enzymes in GSDIa children. FAO enzyme activity is presented as % activity compared to the mean calculated on a pool of healthy subjects.Citrate synthase activity in GSDIa childrenand healthy controls. For all proteins mean value is shown for each group of participants. C1: pediatric controls who had blood collection after the same fasting time as their matched patients. [file 12263_2023_729_MOESM3_ESM.pdf]

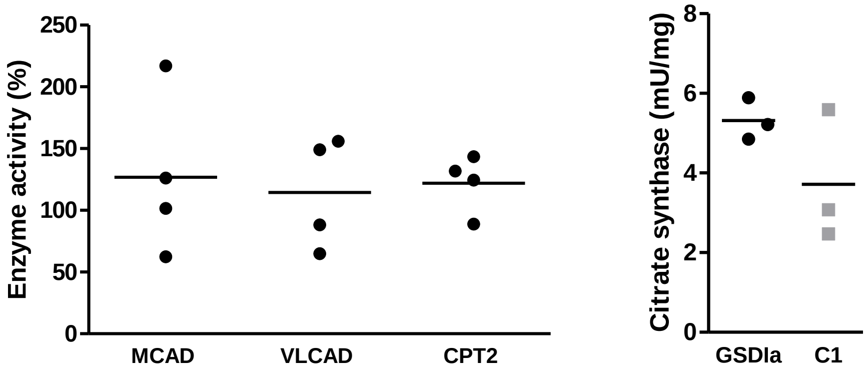

Supplemental figure 3. FAO enzymes (A) and citrate synthase (B) activity in GSDIa children and controls
