# Supplementary material for: Mitochondrial reprogramming in peripheral blood mononuclear cells of patients with glycogen storage disease type Ia
Source: Genes Nutr. 2023 Jun 6;18:10. doi: 10.1186/s12263-023-00729-y (PMC10245432; doi:10.1186/s12263-023-00729-y)
Supplement: Supplementary file 6 — Additional file 6: Supplemental table 1. Clinical information of GSDIa patients. [file 12263_2023_729_MOESM6_ESM.pdf]

**GSDIa subject**	**Age** (years)	**Gender**	**Genotype** (G6PC variants)	**Dietary regimen**	**Carbohydrate intake** (g/kg/day)	**UCCS/Glycosade intake** (g/kg/day)	**Energy intake** (Kcal/kg/day)
			Protein change				
**001**	5.2	M	p.Arg83Cys<br>p.Arg83Cys	Frequent feedings<br>UCCS<br>CNGDF	N.A.	N.A.	N.A.
**002**	10.0	M	p.Arg83Cys<br>p.Arg83Cys	Frequent feedings<br>CNGDF	N.A.	N.A.	N.A.
**003**	17.1	M	p.Trp63Arg<br>p.Arg83Cys	Frequent feedings<br>UCCS	10.5	5.6	46.1
**004**	25.8	F	p.Trp63Arg<br>p.Arg83Cys	Frequent feedings<br>Glycosade	7.1	4.4	31.0
**005**	24.0	F	p.Arg83Cys<br>p.Arg83Cys	Frequent feedings<br>UCCS	6.1	4.0	26.4
**006**	34.9	M	p.Arg83Cys<br>p.Arg83Cys	Frequent feedings<br>Glycosade	6.0	4.6	26.6
**007**	11.2	F	p.Arg83Cys<br>p.Arg83Cys	Frequent feedings<br>UCCS	10.0	4.9	45.1
**008**	28.1	M	p.Arg83Cys<br>p.Arg83Cys	Frequent feedings<br>UCCS<br>CNGDF	4.8	1.4	24.3
**009**	24.1	F	p.Arg83Cys<br>p.Arg83Cys	Frequent feedings<br>UCCS	5.7	4.4	24.5
**010**	11.1	M	p.Arg83Cys<br>p.Arg83Cys	Frequent feedings<br>UCCS	8.3	5.4	34.9

Supplemental table 1. Clinical information of GSDIa patients.
CNGDF: continuous nocturnal gastric drip feeding
N.A.: not available
UCCS: uncooked corn starch
