# Supplementary material for: Mitochondrial reprogramming in peripheral blood mononuclear cells of patients with glycogen storage disease type Ia
Source: Genes Nutr. 2023 Jun 6;18:10. doi: 10.1186/s12263-023-00729-y (PMC10245432; doi:10.1186/s12263-023-00729-y)
Supplement: Supplementary file 7 — Additional file 7: Supplemental table 2. The list of TaqMan assays used for gene expression analysis by real-time quantitative PCR. [file 12263_2023_729_MOESM7_ESM.pdf]

Gene Name	Gene Symbol	Assay ID
**Carnitine Palmitoyltransferase 1A**	CPT1A	Hs00157079_m1
**Succinate Dehydrogenase Complex Flavoprotein Subunit A**	SDHA	HS00188166_m1
**Succinate Dehydrogenase Complex Flavoprotein Subunit B**	SDHB	Hs01042482_m1
**Succinate Dehydrogenase Complex Flavoprotein Subunit C**	SDHC	Hs00818427_m1
**ATP - Citrate Lyase**	ACLY	Hs00982738_m1
**Nicotinamide Phosphoribosyltransferase**	NAMPT	Hs00237184_m1
**Nuclear Respiratory Factor 1**	NRF1	Hs00602161_m1
**Transcription Factor A Mitochondrial**	TFAM	Hs01082775_m1
**Uncoupling Protein 3**	UCP3	Hs01106052_m1
**Peroxisome Proliferative Activated Receptor, Gamma, Coactivator 1, Alpha**	PGC 1α	Hs00173304_m1
**Sirtuin 1**	SIRT1	Hs01009005_m1
**Sirtuin 2**	SIRT2	Hs00247263_m1
**Mechanistic Target Of Rapamycin**	mTOR	Hs00234508_m1
**Akt Serine/Threonine Kinase 1**	AKT1	Hs00178289_m1
**Akt Serine/Threonine Kinase 2**	AKT2	Hs01086102_m1
**Akt Serine/Threonine Kinase 3**	AKT3	Hs00178533_m1
**Beta-2-Microglobulin**	B2M	Hs99999907_m1
**Hypoxanthine Phosphoribosyltransferase 1**	HPRT1	Hs02800695_m1

Supplemental table 2. The list of TaqMan assays used for gene expression analysis by real-time quantitative PCR
